# Diagnostic pathways for lung cancer patients in Denmark: General practice events, first referral and stage at diagnosis

**DOI:** 10.2340/1651-226X.2026.45851

**Published:** 2026-06-02

**Authors:** Dorte Ejg Jarbøl, Peter Haastrup, Sanne Rasmussen, Gitte Bruun Lauridsen, Kirubakaran Balasubramaniam, Linda Juel Ahrenfeldt, Jesper Lykkegaard, Lisa Maria Sele Sætre

**Affiliations:** a Research Unit of General Practice, Department of Public Health, University of Southern Denmark, Odense M, Denmark; b Audit Project Odense, Research Unit of General Practice, University of Southern Denmark, Odense M, Denmark

**Keywords:** lung cancer, diagnosis, evaluation, general practice, symptoms

## Abstract

**Background and purpose:**

This study examined lung cancer patients’ diagnostic pathways, focusing on first healthcare contact, general practice events, referral choice, and stage at diagnosis.

**Patients and methods:**

General practices in three Danish regions participated in a retrospective survey of medical records for patients diagnosed with lung cancer between March 2019 and February 2021. General practitioners completed a questionnaire on initial contact, symptom presentation, diagnostic events and first referral. Socioeconomic data and tumor stage were retrieved from national registers. Associations between symptom presentation and diagnostic events were analysed.

**Results:**

Among 1069 patients, 72% had initial contact in general practice, 22% at a hospital and 5% with out-of-hours care. Of patients starting in general practice (*n* = 766), 36% presented specific cancer symptoms, 48% non-specific symptoms, 9% both and 7% were asymptomatic. Diagnostic events included hesitation to seek care (10%), declining investigation or non-adherence (4%) and initial management for another disease (33%). Men were more likely to decline investigation or fail to follow-up (odds ratio [OR] 4.32; 95% confidence interval 1.75–10.65). Non-specific symptoms increased the odds of referral for another condition (OR 1.48) or another cancer type (OR 2.99). Most patients were first referred to imaging (48%), whereas 16% entered a cancer care pathway and 18% were acutely hospitalised. Advanced stage was common (80%) and more likely among acutely hospitalised patients (OR 2.68).

**Interpretation:**

Most lung cancer patients present first in general practice. Non-specific symptoms are frequent and pose challenges for timely diagnosis.

## Introduction

Timely diagnosis of lung cancer is crucial for both treatment options and survival [1, 2]. However, the diagnostic pathway, from initial symptoms to confirmed diagnosis, is often complex and can be influenced by multiple factors, including the patient’s first point of contact with the healthcare system, the nature of symptom presentation and the clinical management provided by healthcare professionals [3, 4]. Lung cancer screening programmes have been introduced in several countries [5] but have not yet been implemented in Denmark [6]. Despite the screening programmes, most lung cancer cases are still diagnosed based on symptoms presented to general practitioners (GPs) followed by referral for further diagnostic evaluation [4, 7].

Each year about 5000 Danish citizens are diagnosed with lung cancer, making lung cancer the most common cancer in Denmark [2]. Symptoms indicative of lung cancer include specific symptoms such as persisting cough, haemoptysis and dyspnoea as well as non-specific symptoms such as weight loss and tiredness. However, these symptoms are often transient and more commonly associated with benign conditions [8, 9], which makes symptom-based diagnostics a persistent challenge for both patients and clinicians [10]. Symptom presentation at the time of diagnosis has been associated with difference in prognosis [11], although no symptoms, specific symptoms and non-specific symptoms can all occur at all stages of lung cancer [12]. Most studies of cancer diagnostic pathways begin with the initial contact with primary care [4, 13, 14] and therefore overlook the patient interval from symptom onset to consultation [15], which may influence stage at diagnosis and prognosis [16].

As in comparable countries with similar healthcare system structures, Danish GPs are gatekeepers to specialised care. Most diseases are handled in general practice with the possibility of referring patients to other specialists or the hospital for further diagnostic evaluation or treatment when relevant [17]. The organisation emphasises continuity and accessibility, which supports repeated consultations and symptom monitoring over time and places GPs at the core of initial diagnostic processes. During nighttime and weekends, out-of-hours primary care is available for acute conditions [17].

The diagnostic evaluation of cancer is organised in accelerated Cancer Care Pathways (CCPs). When patients aged 40 years or older with a history of smoking present with symp-toms of lung cancer (prolonged cough or change in an existing cough, prolonged hoarseness, dyspnoea or haemop-tysis), Danish GPs are recommended to refer them to diagnostic imaging in the form of computed tomography (CT) of the thorax and upper abdomen [18].

Several factors influence the GP’s suspicion of cancer and subsequent referral decision [19–21]. Nevertheless, most studies on diagnostic pathways fail to incorporate the perspectives and actions taken within general practice, as well as those initiated by the patients prior to consulting the GP [3, 4, 7]. GPs occupy a unique position in the diagnostic pathway, as they are the only healthcare professionals with access to information across the entire course, from initial symptom presentation to final diagnosis, which enables a comprehensive assessment of the diagnostic process [22]. A thorough understanding of the diagnostic pathway and potential delays in lung cancer evaluation is essential to improve the efficiency and accuracy of diagnostics. Therefore, the aim of this study was to examine lung cancer patients’ diagnostic pathways by: (1) identifying the initial point of contact with the healthcare system and (2) analysing associations with possible delaying events, the first referral choice and the Tumor–Node–Metastasis (TNM) stage at diagnosis.

## Patients/material and methods

### Design and settings

In 2021, a survey using the Audit Project Odense method [23] was carried out in the Regions of Southern, Central, and Northern Denmark with the aim of exploring the diagnostic pathways for incident cancer patients. The survey involved GPs conducting a retrospective review of medical records and diagnostic processes for listed patients who had been diagnosed with cancer during a 2-year period. The review was therefore based on already documented clinical information, including consultations, referrals, test results, and diagnostic decisions that had occurred prior to the cancer diagnosis. The invitation process and survey development have been described in detail elsewhere [22].

### Study population

All 852 general practices across the three regions (covering approximately 3.2 million inhabitants (~53% of the population)) were invited to participate in the survey. Patients relevant for the study were identified through the regions’ administrative databases [24] and included all patients diagnosed with incident cancer according to International Classification of Diseases version 10 Cancer Codes C0–C9 (excluding non-melanoma skin cancer C44). The study population included patients diagnosed between 1 March 2019 and 28 February 2021 who were listed with a participating general practice. To ensure that the cancer was incident, patients registered with the same type of cancer in the 5 years prior to the index diagnosis were ineligible. To ensure diagnostic accuracy, the GPs were requested to verify the date of diagnosis and cancer type at the outset of the questionnaire.

This study is one of a series of papers and is based on GP-reported data for lung cancer patients (ICD10, C33, 34) aged 40 years or older in the study population. Detailed information about the data collection procedure can be found elsewhere [22].

### The questionnaire

The questionnaire’s conceptual framework focused on exploring the diagnostic pathway of cancer patients from initial presentation and first contact with the GP to the point of first referral [22].

This study addresses the following constructs among patients diagnosed with lung cancer: place of the patient’s initial contact with symptoms or signs that could have been caused by lung cancer; symptom presentation (specific cancer symptoms (prolonged coughing (4 weeks), dyspnoea, haemoptysis, prolonged hoarseness (> 4 weeks), changes in a familiar cough), non-specific symptoms (weight loss, loss of appetite, fever, fatigue etc.) both, or no symptoms, events in the diagnostic process and first referral for further investigation. (Supplementary Table 1)

### Register data

Data on the patients’ socioeconomic status were obtained by linkage to national registers via the Danish Civil Registration System (CPR) and included the following variables: cohabitation status (single/living alone and married/cohabiting); highest completed level of education (low (< 10 years); medium (10–15 years) and high (> 15 years)); and labour market affiliation (working and out of the workforce (including unemployment and retirement)).

Information about the TNM stage (I–IV (8^th^ edition)) of disease at the time of diagnosis was obtained from the Danish Cancer Registry [25]. Stages I and II were considered local stage disease, whereas stages III and IV were considered advanced stage disease [26].

### Outcome measures

Based on the GP questionnaire, the place of initial contact in the diagnostic pathway was categorised as follows: general practice; out of hours service or another GP than their regular one; hospital, including emergency call and outpatient clinic; and others (another primary care specialist, unknown place of contact or missing).

Events in the diagnostic process were attributed to either the patient or the GP. Patient-related events included hesitation to contact the GP; reluctance to undergo investigation; and non-compliance with agreed follow-up care. GP-related events comprised recommending watchful waiting without a time-frame; initial treatment or referral based on suspicion of another disease; postponement of further investigations due to normal examination; and initial referral for investigation of a different cancer type.

The initial referral from general practice was categorised into the one of the following pathways: CCP; Non-Specific Symptoms and Signs of Cancer-Cancer Care Pathway (NSSC-CCP); diagnostic imaging; referral to a medical specialist or outpatient clinic; and acute hospitalisation.

All variables used in the study, with the exact wording, response scales, variable coding and data sources, are available in Supplementary Table 2.

### Statistical analyses

Firstly, we explored initial contact to the healthcare system among all lung cancer patients aged 40 years or older. Secondly, we investigated the outcome variables ‘events in the diagnostic process’ and ‘first referral’ among the patients whose initial contact was in general practice.

Associations between sex, age and symptom presentation and events in the diagnostic process and in the first referral were analysed using logistic regression analyses with robust standard errors clustered by each general practice, adjusted for sex, age, symptom presentation and socioeconomic status.

Associations between sex, age, symptom presentation, events in the diagnostic process, first referral and TNM stage at diagnosis were analysed using the same methods as described above.

## Results

**Figure 1:**
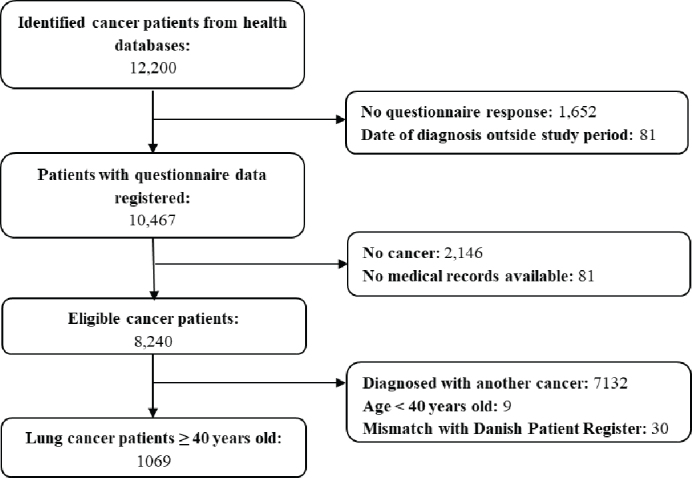
Flowchart.

A total of 187 general practices (21%) participated in the study. From the regions’ administrative databases, a total of 12,200 of the patients listed with the participating practices were registered with an incident cancer diagnosis received during the study period. The GPs completed the questionnaire for 10,467 of the cancer patients. Patients who did not have cancer or available medical records were excluded, which resulted in a sample of 8240 patients with cancer, of whom 1069 were 40 years or older and were diagnosed with lung cancer, see Figure 1.

Table 1 provides characteristics of the study population stratified based on the place of the initial contact. A total of 766 patients (72%) had their initial contact in general practice, 232 (22%) with a hospital, 48 (5%) with the out-of-hours primary care services, and 23 (2%) were diagnosed through other pathways, including a primary care specialist, unknown sources or missing data.

**Table 1:** Characteristics of lung cancer patients above 40 years stratified on place of initial contact with symptoms attributable to the later diagnosed lung cancer (*N* = 1069).

Variables	General practice	Out of hours service	Hospital, including emergency call/112, outpatient clinic	Others (primary care specialist, unknown, missing)
	*n* (%)	*n* (%)	*n* (%)	*n* (%)
**Total**	766 (71.6)	48 (4.5)	232 (21.7)	23 (2.1)
**Sex**				
Women	400 (52.2)	21 (43.8)	114 (49.1)	-
Men	366 (47.8)	27 (56.3)	118 (50.9)	-
**Age groups (years)**				
40–59	104 (13.6)	5 (10.4)	22 (9.5)	-
60–79	546 (71.3)	30 (62.5)	165 (71.1)	-
80+	116 (15.1)	13 (27.1)	45 (19.4)	-
**Region**				
Region of Southern Denmark	113 (14.8)	8 (16.7)	23 (9.9)	-
The Central Denmark Region	365 (47.7)	24 (50.0)	114 (49.1)	-
The North Denmark Region	288 (37.6)	16 (33.3)	95 (40.9)	-
**Cohabitation status**				
Single/living alone	318 (41.5)	26 (54.2)	104 (44.8)	-
Married/cohabiting	448 (58.5)	22 (45.8)	128 (55.2)	-
**Highest obtained level of education**				
Low (< 10 years)	185 (24.2)	17 (35.4)	76 (32.8)	-
Medium (10–15 years)	479 (62.5)	31 (64.6)	131 (56.5)	-
High (> 15 years)	102 (13.3)	-*	25 (10.8)	-
**Labour market affiliation**				
Working	134 (17.5)	5 (10.4)	21 (9.1)	-
Out of the workforce	632 (82.5)	43 (89.6)	211 (90.9)	-

* Within that category, the values below five have been added to the nearest cell.

Table 2 displays diagnostic events among the 766 lung cancer patients with initial contact in general practice, including adjusted associations with symptom presentation, sex and age.

**Table 2:** Adjusted odds ratios^1^ (OR), 95% confidence interval (CIs) for events in the diagnostic process according to age, sex and symptom presentation for the patients with lung cancer above 40 years with initial contact in Danish general practice (*N* = 766).

Variables	All	The patient hesitated to contact the GP	The patient was reluctant to undergo investigation and non-compliant with agreed follow-up care	The GP advised watchful waiting without a time frame	The GP initially treated or referred on the suspicion of another disease	The GP waited with further investigation because of a normal examination	The GP initially referred to investigation of another cancer type than lung cancer	None
	*n* (%)	*n* (%)	*n* (%)	*n* (%)	*n* (%)	*n* (%)	*n* (%)	*n* (%)
Total*	766 (100%)	77 (10.1)	28 (3.7)	21 (2.7)	251 (32.8)	50 (6.5)	45 (5.9)	383 (50.0)
		**Adj. OR (95% CI)**	**Adj. OR (95% CI)**	**Adj. OR (95% CI)**	**Adj. OR (95% CI)**	**Adj. OR (95% CI)**	**Adj. OR (95% CI)**	**Adj. OR (95% CI)**
**Sex**								
Women	400 (52.2)	Ref	Ref	Ref	Ref	Ref	Ref	Ref
Men	366 (47.8)	1.12 (0.67–1.85)	4.32 (1.75–10.65)	0.66 (0.27–1.61)	0.76 (0.55–1.05)	0.70 (0.38–1.29)	0.74 (0.39–1.42)	1.14 (0.84–1.55)
**Age groups, years**								
40–59	104 (13.6)	Ref	Ref	Ref	Ref	Ref	Ref	Ref
60–79	546 (71.3)	1.08 (0.44–2.65)	0.50 (0.22–1.14)	0.89 (0.24–3.33)	1.32 (0.75–2.35)	1.17 (0.40–3.40)	0.84 (0.38–1.84)	0.94 (0.56–1.59)
≥ 80	116 (15.1)	0.87 (0.30–2.55)	-	3.37 (0.66–17.16)	1.74 (0.87–3.48)	1.68 (0.46–6.10)	0.95 (0.31–2.90)	0.71 (0.38–1.35)
**Symptom presentation**								
Specific cancer symptoms	277 (36.2)	Ref	Ref	Ref	Ref	Ref	Ref	Ref
Non-specific symptoms	364 (47.5)	0.81 (0.48–1.39)	1.83 (0.68–4.93)	0.55 (0.21–1.42)	1.48 (1.05–2.07)	0.68 (0.36–1.26)	2.99 (1.24–7.20)	0.73 (0.53–1.00)
Both	66 (8.6)	2.24 (1.11–4.53)	3.45 (1.03–11.59)	0.39 (0.05–3.15)	1.14 (0.63–2.07)	0.95 (0.34–2.65)	6.16 (2.07–18.35)	0.48 (0.28–0.84)
None	59 (7.7)	0.29 (0.07–1.23)	0.79 (0.08–7.44)	0.43 (0.06–3.22)	0.61 (0.31–1.19)	0.19 (0.02–1.47)	3.31 (1.04–10.59)	1.82 (1.01–3.30)

1 Adjusted for age, sex, symptom presentation, cohabitation status, labour market affiliation and educational level.

* The number of the different events does not sum up to 100% as it was also possible to indicate none of the abovementioned events.

For 77 patients (10%), hesitation to contact the GP was reported, whereas 28 patients (4%) either were reluctant to undergo investigation or non-compliant with agreed follow-up care. Compared to women, men had higher odds of not wanting an investigation or not adhering to the follow-up agreement (odds ratio [OR] 4.32; 95% confidence interval [CI] 1.75–10.65).

For 251 of the patients (33%), the GP initially treated or referred them on suspicion of another disease; for 45 (5.9%) patients, the GP initially referred them to investigation for another cancer type than lung cancer; and for 50 patients (7%), further investigation was postponed due to normal examination results. These events seemed less frequent among men than women, although the differences were not statistically significant.

One third of the patients (36%) presented with specific cancer symptoms, 48% with non-specific symptoms, 9% with a combination of both and 7% were asymptomatic at the time of initial contact. Compared to patients presenting with specific cancer symptoms, those with non-specific symptoms had increased odds of being initially treated or referred on suspicion of another disease (OR 1.48; 95% CI 1.05–2.07) and of being referred for investigation of a different cancer type (OR 2.99; 95% CI 1.24–7.20). They also had reduced odds of experiencing no diagnostic events (OR 0.73; 95% CI 0.53–1.00). In contrast, patients presenting without symptoms had higher odds of no diagnostic events (OR 1.82; 95% CI 1.01–3.30). Compared to patients presenting only with specific cancer symptoms, patients presenting with both specific and non-specific symp-toms had increased odds of having hesitated to seek healthcare (OR 2.24; 95% CI 1.11–4.53), of being reluctant to undergo investigation or non-compliant with agreed follow-up care (OR 3.45; 95% CI 1.03–11.59), and of being initially referred for investigation of a different cancer type (OR 6.16; 95% CI 2.07–18.35) (Table 2).

Table 3 shows the associations between sex, age, symptom presentation and the first referral in the diagnostic pathway for the patients with initial contact in general practice*.* A majority of the patients (391, 48%) were first referred to diagnostic imaging, whereas 124 patients (16%) were referred to a specific CCP, 40 patients (5%) were referred to a NSSC-CCP and 136 patients (18%) were acutely hospitalised. No statistically significant associations were found for sex and age; however, the oldest age group tended to be less likely to be referred to CCP and NSSC-CCP and more likely to undergo acute hospitalisation.

**Table 3:** Results of the multivariable logistic regression models estimating the associations between age, sex and symptom presentation and the first referral in the diagnostic pathway, shown as adjusted odds ratios^1^ (Adj. ORs) with 95% confidence intervals (CIs) (*N* = 766).

Variables	First referral in the diagnostic pathway				
	Cancer care pathway	Non-specific symptoms and signs of cancer care pathway	Diagnostic imaging	Specialist or outpatient clinic	Acute hospitalisation
**Total** *	124 (16.2)	40 (5.2)	391 (51.0)	75 (9.8)	136 (17.8)
	**Adj. OR (95% CI)**	**Adj. OR (95% CI)**	**Adj. OR (95% CI)**	**Adj. OR (95% CI)**	**Adj. OR (95% CI)**
**Sex**					
Women	Ref	Ref	Ref	Ref	Ref
Men	1.19 (0.80–1.79)	1.46 (0.76–2.80)	0.79 (0.58–1.06)	0.75 (0.46–1.24)	1.35 (0.90–2.02)
**Age groups, years**					
40–59	Ref	Ref	Ref	Ref	Ref
60–79	0.58 (0.31–1.10)	0.75 (0.27–2.11)	1.68 (1.01–2.81)	0.66 (0.31–1.39)	1.10 (0.53–2.27)
≥ 80	0.49 (0.21–1.12)	0.41 (0.11–1.58)	1.53 (0.82–2.86)	0.99 (0.39–2.49)	1.38 (0.59–3.20)
**Symptom presentation**					
None	0.59 (0.26–1.33)	1.00 (1.00–1.00)	0.95 (0.54–1.68)	1.15 (0.45–2.98)	1.89 (0.92–3.88)
Specific cancer symptoms	Ref	Ref	Ref	Ref	Ref
Non-specific symptoms	0.51 (0.33–0.79)	7.22 (2.55–20.49)	0.72 (0.52–0.99)	1.25 (0.73–2.12)	1.68 (1.09–2.58)
Both	0.94 (0.47–1.85)	1.02 (0.11–9.20)	1.16 (0.67–2.01)	0.67 (0.22–2.01)	1.04 (0.49–2.20)

1 Adjusted for age, sex, symptom presentation, cohabitation status, labour market affiliation and educational level.

* The number of the different processes does not add up to 100% as respondents also had the option to indicate none of the above-mentioned processes.

In the analyses of symptom presentation, patients with non-specific symptoms were more likely to be referred to a NSSC-CCP (OR 7.22; 95% CI 2.55–20.59) and to acute hospitalisation (OR 1.68; 95% CI 1.09–2.58) compared to patients with specific cancer symptoms (Table 3).

Table 4 shows the stage distribution among the patients with initial contact in general practice and a registered TNM stage in the register and the associations between sex, age, symptom presentation, GP events, diagnostic pathway and the stage at diagnosis (*n* = 615). Most patients were diagnosed in advanced stage (493, 80%). Stage at diagnosis was equally distributed across sex, age and symptom presentation. Compared to patients referred through a CCP, patients who were acutely hospitalised were more likely to be diagnosed with advanced stage cancer (OR 2.68, 95% CI 1.21–5.92).

**Table 4:** Associations between sex, age, symptom presentation, events in the diagnostic process, the first referral and stage at diagnosis among lung cancer patients presenting in general practice (*N* = 615).

Variables		Total	Stage at diagnosis		
			Local TNM stage I/II	Advanced TNM stage III/IV	Local vs. advanced stage
		*N* (%)	*n* (%)	*n* (%)	OR (95% CI)
Total*		615 (100.0)	122 (19.8)	493 (80.2)	-
**Sex**					
Women		336 (54.6)	77 (63.1)	259 (52.5)	ref
Men		279 (45.4)	45 (36.9)	234 (47.5)	1.35 (0.86–2.12)
**Age group**					
40–59 years		74 (12.0)	12 (9.8)	62 (12.6)	ref
60–79 years		431 (70.1)	91 (74.6)	340 (69.0)	0.71 (0.33–1.51)
≥ 80 years		110 (17.9)	19 (15.6)	91 (18.5)	0.93 (0.37–2.32)
**Symptom presentation**					
None		47 (7.6)	13 (10.7)	34 (6.9)	0.80 (0.38–1.69)
Specific cancer symptoms		228 (37.1)	49 (40.2)	179 (36.3)	ref
Non-specific symptoms		296 (48.1)	60 (49.2)*	236 (47.9)	1.14 (0.72–1.81)
Both		44 (7.2)	< 5	44 (8.9)	2.93 (0.99–8.62)
**Events in the diagnostic process Yes/no (ref)**					
The patient hesitated to contact the GP		62 (10.1)	11 (9.0)	51 (10.3)	0.66 (0.24–1.82)
The patient did not want an investigation/did not comply with follow-up agreement		21 (3.4)	< 5	21 (4.3)	4.21 (0.41–43.48)
The GP initially treated or referred on the suspicion of another disease		205 (33.3)	34 (27.9)	171 (34.7)	0.85 (0.32–2.27)
The GP waited with further investigation because of a normal examination		38 (6.2)	6 (4.9)	32 (6.5)	0.92 (0.31–2.68)
The GP initially referred to investigation of another type of cancer than lung cancer		35 (5.7)	8 (6.6)	27 (5.5)	0.64 (0.22; 1.83)
**Diagnostic pathway Yes/no**					
**Referred to**	Cancer care pathway	105 (17.1)	26 (21.3)	79 (16.0)	ref
	Patient Pathway for Non-specific Symptoms and Signs of Cancer	29 (4.7)	7 (5.7)	22 (4.5)	1.05 (0.39; 2.82)
	Diagnostic imaging	310 (50.4)	59 (48.4)	251 (50.9)	1.49 (0.85; 2.59)
	Specialist or hospital	65 (10.6)	18 (14.8)	47 (9.5)	0.73 (0.35; 1.52)
	Acute hospitalisation	106 (17.2)	12 (9.8)	94 (19.1)	2.68 (1.21; 5.92)

* Within that category, the values below five have been added to the nearest cell.

Adjusted for age, sex, symptom presentation, cohabitation status, labour market affiliation and educational level.

*N* differ from in this Table, because stage at diagnosis were not registered for all patients. GP: general practitioners.

## Discussion and conclusion

### Main findings

A majority of the lung cancer patients initiated their diagnostic pathway in general practice, whereas approximately one in five had their initial contact within a hospital setting. Among the patients whose diagnostic pathway began in general practice, two thirds were first referred for diagnostic imaging or to a CCP, in accordance with national guidelines. Most of the patients presented with non-specific symptoms in general practice, whereas one third presented with symptoms specific to lung cancer. Presentation with non-specific symptoms was associated with a higher likelihood of referral to a NSSC-CCP and acute hospitalisation.

The most common diagnostic event was the GP treating or referring the patient based on suspicion of another disease, a pattern more frequently observed among patients presenting with non-specific symptoms. A combination of specific and non-specific symptoms more than doubled the likelihood of patient-related events, including hesitation to seek healthcare, reluctance to undergo diagnostic investigation and non-compliance with follow-up agreements. Furthermore, this combination of symptoms increased the likelihood of the GP referring the patient for investigation of another cancer type by a factor of six.

Acute hospitalisation was associated with a higher likelihood of advanced stage disease, whereas none of the streamlined pathways to diagnosis were associated with TNM stage at diagnosis.

### Discussion of results and comparison with existing literature

In the present study, most of the lung cancer patients had their first contact in general practice, which is in line with the findings of a previous Danish study investigating the GP’s involvement in lung cancer diagnostics [7]. Half of the patients who initiated their diagnostic pathway in general practice were referred for diagnostic imaging, whereas 16% were referred to a lung CCP. Both options could be considered good clinical practice in accordance with the Danish lung CCP. Christensen et al. found that 38% of lung cancer patients were referred for a CCP from primary care, whereas a third are referred from secondary care [4]. The difference between these results and the present study might be due to methodological differences, since Christensen et al. based their findings on register data [4]. The first step of the Danish lung CCP is referral for diagnostic imaging, and the CCP will only continue at the department of pulmonology if the imaging confirms suspicion of lung cancer [18]. Hence, some GPs in our study may have answered that they referred the patient to diagnostic imaging rather than to a CCP. In a survey-based study, Jensen et al. also found that most lung cancer patients initiated their diagnostic pathway in general practice, and that around 41% of the patients were referred to the lung CCP [27]. This proportion is considerably higher than that reported in the present study. However, referral options and clinical guidelines have evolved since the publication of Jensen et al.’s study [27]. Consequently, the findings of the present study provide a more current and nuanced depiction of diagnostic pathways under the updated guidelines.

Regardless of stage at diagnosis, approximately 90% of the patients had presented at least one symptom to their GP. This percentage is higher than that reported in Ruano-Ravina et al. [12] but in line with Koo et al., who claimed that symptom-based timely diagnosis may be feasible, although challenging [28]. Studies have shown that most localised lung cancers are detected incidentally [29], suggesting that symptom‑based diagnostic approaches alone may not be sufficient to ensure timely diagnosis of lung cancer. Therefore, other diagnostically feasible strategies, such as screening, are also likely to be beneficial [30].

As expected, we found that non-specific symptom presentation increased the likelihood of referral to NSSC-CCP and lowered the odds of referral to a CCP. These findings are in line with a systematic review which concluded that lung cancer is among the most common cancers diagnosed through the NSSC-CCPs [31].

We also found that the combination of non-specific and specific symptoms was associated with both the patients’ behaviour and the GPs’ referrals. Patients more often hesitated to seek healthcare, which is in line with studies on healthcare-seeking behaviour [32, 33] and barriers to GP contact [34, 35]. In the present study, the GPs more often referred patients with both specific and non-specific symptoms to investigation for another cancer type than lung cancer. This highlights the ongoing challenges in interpreting lung cancer symptoms and determining when to raise clinical suspicion, which have also been documented in previous studies [36, 37], underlining a need for paying attention to this group. Patients often interpret their symptoms in relation to known causes, such as smoking or pre-existing diseases [32, 38], and the presence of multiple symptoms may lead some patients to rationalise or dismiss their experiences, thereby delaying the decision to seek medical attention [14, 39]. Likewise, it may serve as a diagnostic bias for the GPs [40].

The percentage of patients who initiated their lung cancer diagnostic pathway with acute hospitalisation was higher than that reported in Guldbrandt et al. [7] but in line with studies from the UK [41]. However, the definitions of emergency presentation vary within the existing literature, which makes comparison challenging [41]. This may indicate that some patients delay seeking medical attention for lung cancer symptoms until the symptoms have become sufficiently pronounced to necessitate hospital admission [34, 35]. Providing the characteristics of the group of patients diagnosed through emergency presentation may guide interventions targeting these patients and could reduce some of the inequities in lung cancer survival. For instance, challenged health literacy, a current smoking history or previous bad experiences with the healthcare system may compromise healthcare-seeking behaviour and diagnostic evaluation, thereby increasing the risk of emergency presentation and advanced stage diagnosis [35, 42, 43]. Further, the present study confirmed associations between non-specific symptoms, acute hospitalisation and advanced stage diagnosis, which emphasises that there is still a need to improve the chances of timely diagnosis based on non-specific symptoms.

### Strengths and limitations

The inclusion of patients in the survey was based on the regions’ administrative databases, which in general have high completeness [25]. To further validate the cancer diagnoses, GPs verified the date and diagnosis for each patient based on their medical records, and some discrepancies were identified. The number of patients with lung cancer included corresponds to the expected number taken the lung cancer incidence and GP participation rates in account, confirming the validity of the final dataset. Furthermore, the survey data were linked with registry data from Statistics Denmark, which enabled access to detailed information on the patients’ socioeconomic status and cancer stage. Unfortunately, the data did not include information about existing respiratory diseases or smoking status, inducing a risk of residual confounding.

The low participation rate among eligible GPs (21%) introduces a potential selection bias. On the one hand, the participating GPs may have had a particular interest in cancer diagnostics, which may have led to an overrepresentation of CCPs and more favourable assessments of the diagnostic process. On the other hand, GPs who find cancer diagnosis challenging may have been motivated to participate with the aim of seeking insight or improvement, which may have skewed the data in the opposite direction. Therefore, the direction and magnitude of a potential selection bias remain uncertain. Moreover, the TNM classification was available only for 615 of the 766 patients. However, the distribution of the explanatory variables did not differ between the 766 and the 615 patients, as it appears from the absolute numbers for age, sex, symptom presentation and events in general practice.

Questionnaires were pilot tested to ensure content validity. As they relied solely on information recorded in patients’ medical files or recalled by GPs, underreporting of diagnostic events cannot be ruled out. Diagnostic events were documented for only half of the patients, which may reflect fewer complications but could also indicate unreported contributing factors or missed diagnostic opportunities. The completion period, ranging from a few weeks to 2 years after the cancer diagnosis, introduces a risk of recall bias and misclassification. Although analyses were adjusted for known confounders, chronic respi-ratory conditions and smoking may represent residual confounding.

Having GPs assess their own patients’ medical records may be a limitation, as their evaluations can be influenced by existing patient relationships and knowledge of outcomes. Moreover, behavioural factors such as hesitation, reluctance, or non-compliance are often undocumented and may therefore remain unreported.

The specific imaging modality reported by GPs is unknown. Distinguishing between chest X‑ray and thoracic CT would have offered important insight into whether GPs follow the diagnostic recommendations of the Danish CCP.

## Implications

The diagnostic pathway of lung cancer is challenged by patients presenting outside general practice and thus in a pathway that is parallel to the established CCPs. As most lung cancer patients are elderly and have a history of smoking, it may be important for GPs to provide education and build a positive relationship with these patients in order to reduce barriers to seeking medical attention when new respiratory symptoms or changes in existing symptoms arise.

The symptom-based diagnostic approach is a continuous challenge. Screening may be helpful, but it is also necessary to improve the efficiency and opportunities for diagnostics in general practice. One recent study has suggested using artificial intelligence to analyse medical records as an opportunity to diagnose lung cancer earlier [44]. Other prospects include risk assessment tools in general practice and possibly biochemical tests [45, 46].

Every third lung cancer patient presenting in primary care was initially treated for or referred on suspicion of another disease. This indicates a continuous need for disseminating sufficient knowledge about cancer symptoms and choice of referral to the GPs and for ensuring that the access to diagnostic tools is adequate [47].

## Conclusion

General practice is most often the initial point of contact for patients diagnosed with lung cancer, although every fifth lung cancer patient is diagnosed based on initial contact within the hospital; this is most noticeable among the oldest patients. More than 90% of the patients presented with at least one symptom, and non-specific symptoms were associated with a higher likelihood of the patients having hesitated to seek healthcare, of referral for a NSSC-CCP, of acute hospitalisation and of advanced stage diagnosis. No associations were found between pathways to diagnosis and stage at diagnosis.

## Supplementary Material



## Data Availability

The datasets generated and analysed for the current study are not publicly available and cannot be shared due to the data protection regulations enacted by the Danish Data Protection Agency. Access to data is strictly limited to the researchers who have obtained permission for data processing. This permission was granted to the Research Unit of General Practice, Department of Public Health, University of Southern Denmark. Further inquiries can be made to PI Jesper Lykkegaard (email: jlykkegaard@health.sdu.dk)
