# Supplementary material for: Diagnostic pathways for lung cancer patients in Denmark: General practice events, first referral and stage at diagnosis
Source: Acta Oncol. 2026 Jun 2;65:45851. doi: 10.2340/1651-226X.2026.45851 (PMC13234914; doi:10.2340/1651-226X.2026.45851)
Supplement: Supplementary file 1 [file AO-65-45851-s1.pdf]

Supplementary material has been published as submitted. It has not been copyedited, or typeset by Acta Oncologica

Supplementary Table s1: Symptom list

**Non-specific symptoms (general alarming symptoms or signs)**<br><br>Symptoms or signs that may be due to serious illness but which in themselves do not prompt an organ-specific cancer patient pathway<br><br>Frequent, general symptoms, that on their own do not cause suspicion of serious illness	Weight loss<br>Loss of appetite<br>Fever without known origin<br>Night sweats<br>Increased tendency of infection<br>Swollen lymph node<br>Anaemia<br>Fatigue<br>Nausea<br>Bloating/abdominal distention<br>Concentration difficulty<br>Memory difficulty<br>Malaise<br>Discomfort<br>Non-specific pain<br>Lack of energy<br>Vertigo<br>Headache<br>Back pain<br>Swollen legs
**Specific lung cancer symptoms and signs:**<br>Symptoms and signs mentioned in organ-specific cancer patient pathway that prompt a cancer patient pathway	**Age > 40 years with a relevant history of smoking:**<br>Prolonged coughing (4 weeks)<br>Dyspnoea<br>Hemoptysis<br>Prolonged hoarseness (>4 weeks)<br>Changes in a familiar cough

Supplementary Table 2: Codebook

Variable	Category	Data type	Items	Response categories and labels
**Events in the diagnostic course**	Patient hesitated	Reported by the GP (by survey answer)	The patient described that he/she had hesitated to see a GP	No (0), Yes (1)
	No diagnostic investigation wanted	Reported by the GP (by survey answer)	The patient did not want a diagnostic investigation	No (0), Yes (1)
	No follow-up	Reported by the GP (by survey answer)	The patient did not comply with the follow-up agreement	No (0), Yes (1)
	Wait and see	Reported by the GP (by survey answer)	The GP advised to wait and see without a time indication	No (0), Yes (1)
	Treatment or referral for another illness	Reported by the GP (by survey answer)	The GP treated or referred on suspicion of another illness than cancer first	No (0), Yes (1)
	Normal tests	Reported by the GP (by survey answer)	The GP waited because of normal test results	No (0), Yes (1)
	Suspicion of another cancer type	Reported by the GP (by survey answer)	The GP referred the patient on suspicion of another cancer type first	No (0), Yes (1)
**The GP's initial referral**	Cancer patient pathway	Reported by the GP (by survey answer)	Referred to specific cancer patient pathway first	No (0), Yes (1)
	Non-specific Cancer Patient Pathway	Reported by the GP (by survey answer)	Referred to a Diagnostic Center (in a non-specific signs and symptoms of cancer-cancer patient pathway) first	No (0), Yes (1)
	Diagnostic imaging	Reported by the GP (by survey answer)	Referred to diagnostic imaging first	No (0), Yes (1)
	Specialist or another hospital department	Reported by the GP (by survey answer)	Referred to a specialist or another hospital department first	No (0), Yes (1)
	Acute hospitalization	Reported by the GP (by survey answer)	Acutely hospitalized	No (0), Yes (1)
**Initial place of contact**	Out of hours service	Reported by the GP (by survey answer)	Where did the patient first turn to with symptoms or signs that you retrospectively believe could be due to the cancer: the	No (0), Yes (1)

			out of hours service or another general practitioner than their regular	
	Specialist	Reported by the GP (by survey answer)	Where did the patient first turn to with symptoms or signs that you retrospectively believe could be due to the cancer: a medical specialist (other than a general practitioner)	No (0), Yes (1)
	Hospital	Reported by the GP (by survey answer)	Where did the patient first turn to with symptoms or signs that you retrospectively believe could be due to the cancer: the hospital, including emergency call/112, outpatient clinic and during hospitalization	No (0), Yes (1)
	No symptoms	Reported by the GP (by survey answer)	No symptoms. The cancer was diagnosed based on screening (breast, colorectal or cervix)	No (0), Yes (1)
	Unknown	Reported by the GP (by survey answer)	Unknown where the patient was first seen	No (0), Yes (1)
	General practitioner	Reported by the GP (by survey answer)	Where did the patient first turn to with symptoms or signs that you retrospectively believe could be due to the cancer: their own general practitioner	No (0), Yes (1)
**Explanatory variables:**				
**Patient characteristics**	Gender	Determined from the patient's civil registration number	Female or male	Female (0), Male (1)
	Age	Determined from the patient's civil registration number	Age at the time of the cancer diagnosis	Continuous<br>For analyses categorized:<br>0-20, 21-40, 41-60, 61-80 and >80
**First symptoms that could be due to the cancer**	Non-specific or general symptoms	Reported by the GP (by survey answer)	Non-specific or general symptoms as the presenting symptoms or signs that retrospectively could be due to the cancer	No (0), Yes (1)
	Specific alarm symptoms	Reported by the GP (by survey answer)	Specific cancer symptoms as the presenting symptoms or signs that retrospectively could be due to the cancer	No (0), Yes (1)
	None	Reported by the GP (by survey answer)	No symptoms as the presenting symptoms or signs that retrospectively could be due to the cancer (was diagnosed by a coincident	No (0), Yes (1)

			finding in the examination, blood tests or diagnostic imaging)	
	Do not know	Reported by the GP (by survey answer)	Unknown which symptoms were the presenting symptoms or signs that retrospectively could be due to the cancer	No (0), Yes (1)
**Exclusion criteria**		Reported by the GP (by survey answer)	The patient did not have a new cancer diagnosis at the time of diagnosis stated	No (0), Yes (1)
		Reported by the GP (by survey answer)	Do not have any patient records	No (0), Yes (1)
		**Register data**		
**Cohabitation status**		Register of Family and Income		Single/living alone<br>Married/cohabiting
**Highest obtained level of education**		Danish Education Register		Low (< 10 years)<br>Medium (10-15 years)<br>High (>15 years)
**Labour market affiliation**		Income Statistics Register		Working<br>Out of the workforce
**Tumour Nodal Metastasis (TNM) stage**		Danish Cancer Registry		Local stage: TNM Stage I and II<br>Advanced stage: TNM Stage III and IV
